# Converting PROMIS^®^-29 v2.0 profile data to SF-36 physical and mental component summary scores in patients with cardiovascular disorders

**DOI:** 10.1186/s12955-024-02277-4

**Published:** 2024-08-15

**Authors:** Gregor Liegl, Felix H. Fischer, Carl N. Martin, Maria Rönnefarth, Annelie Blumrich, Michael Ahmadi, Leif-Hendrik Boldt, Kai-Uwe Eckardt, Matthias Endres, Frank Edelmann, Holger Gerhardt, Ulrike Grittner, Arash Haghikia, Norbert Hübner, Ulf Landmesser, David Leistner, Knut Mai, Jil Kollmus-Heege, Dominik N. Müller, Christian H. Nolte, Sophie K. Piper, Kai M. Schmidt-Ott, Tobias Pischon, Simrit Rattan, Ira Rohrpasser-Napierkowski, Katharina Schönrath, Jeanette Schulz-Menger, Oliver Schweizerhof, Joachim Spranger, Joachim E. Weber, Martin Witzenrath, Sein Schmidt, Matthias Rose

**Affiliations:** 1 Department of Psychosomatic Medicine, Center for Patient-Centered Outcomes Research, Charité – Universitätsmedizin Berlin, corporate member of Freie Universität Berlin and Humboldt- Universität zu Berlin, Charitéplatz 1, 10117 Berlin, Germany; 2 Berlin Institute of Health at Charité – Universitätsmedizin Berlin, Berlin, Germany; 3 Center for Stroke Research Berlin, Charité - Universitätsmedizin Berlin, corporate member of Freie Universität Berlin and Humboldt- Universität zu Berlin, Berlin, Germany; 4 Department of Neurology, Charité - Universitätsmedizin Berlin, corporate member of Freie Universität Berlin and Humboldt- Universität zu Berlin, Berlin, Germany; 5 Department of Nephrology and Medical Intensive Care, Charité - Universitätsmedizin Berlin, corporate member of Freie Universität Berlin and Humboldt- Universität zu Berlin, Berlin, Germany; 6 Department of Internal Medicine and Cardiology, Charité - Universitätsmedizin Berlin, corporate member of Freie Universität Berlin and Humboldt- Universität zu Berlin, Berlin, Germany; 7 German Centre for Cardiovascular Research (DZHK), partner site Berlin, Berlin, Germany; 8 German Center for Neurodegenerative Diseases (DZNE), partner site Berlin, Berlin, Germany; 9 Exellence Cluster NeuroCure, Berlin, Germany; 10 Max Delbrück Center for Molecular Medicine in the Helmholtz Association (MDC), Berlin, Germany; 11 Institute of Biometry and Clinical Epidemiology, Charité - Universitätsmedizin Berlin, corporate member of Freie Universität Berlin and Humboldt- Universität zu Berlin, Berlin, Germany; 12 ECRC, Department of Cardiology, Charité - Universitätsmedizin Berlin, corporate member of Freie Universität Berlin and Humboldt- Universität zu Berlin, Berlin, Germany; 13 Charité - Universitätsmedizin Berlin, corporate member of Freie Universität Berlin and Humboldt- Universität zu Berlin, Berlin, Germany; 14 Department of Cardiology, Angiology and Intensive Care Medicine, Deutsches Herzzentrum der Charité (DHZC), Berlin, Germany; 15 Department of Cardiology, Charité - Universitätsmedizin Berlin, corporate member of Freie Universität Berlin and Humboldt- Universität zu Berlin, Berlin, Germany; 16 Department of Endocrinology & Metabolism, Charité - Universitätsmedizin Berlin, corporate member of Freie Universität Berlin and Humboldt- Universität zu Berlin, Berlin, Germany; 17 Charité - Universitätsmedizin Berlin, corporate member of Freie Universität Berlin and Humboldt- Universität zu Berlin, Charité-Center for Cardiovascular Research (CCR), Berlin, Germany; 18 Experimental and Clinical Research Center (ECRC), a cooperation of Charité - Universitätsmedizin Berlin and Max Delbruck Center for Molecular Medicine (MDC), Berlin, Germany; 19 Division of Pulmonary Inflammation, Department of Infectious Diseases and Respiratory Medicine, Charité – Universitätsmedizin Berlin, corporate member of Freie Universität Berlin and Humboldt- Universität zu Berlin, Charitéplatz 1, 10117 Berlin, Germany; 20 German Center for Lung Research (DZL), Giessen, Germany; 21 Molecular Epidemiology Research Group, Max Delbrück Center for Molecular Medicine in the Helmholtz Association (MDC), Berlin, Germany; 22 Max Delbrück Center for Molecular Medicine in the Helmholtz Association (MDC), Biobank Technology Platform, Berlin, Germany; 23 German Center for Diabetes Research, München-Neuherberg, Germany; 24 Institute of Medical Informatics, Charité - Universitätsmedizin Berlin, corporate member of Freie Universität Berlin and Humboldt- Universität zu Berlin, Berlin, Germany; 25 Department of Nephrology and Hypertension, Hannover Medical School, Hannover, Germany; 26 Clinic for Psychosomatic Medicine and Psychotherapy, University of Duisburg-Essen, LVR-University Hospital Essen, Essen, Germany; 27 Centre for Translational Neuro- and Behavioral Sciences (C-TNBS), University of Duisburg-Essen, Essen, Germany; 28 Department of Medicine, Cardiology, Goethe University Hospital, Frankfurt, Germany; 29 German Center for Cardiovascular Research (DZHK) Partner Site RheinMain, Frankfurt, Germany

**Keywords:** PROMIS-29, SF-36, Health composite scores, Cardiovascular diseases, Mapping, Patient-reported outcomes, Outcome measures, Health-related quality of life

## Abstract

**Background:**

Health-related quality of life (HRQL) has become an important outcome parameter in cardiology. The MOS 36-ltem Short-Form Health Survey (SF-36) and the PROMIS-29 are two widely used generic measures providing composite HRQL scores. The domains of the SF-36, a well-established instrument utilized for several decades, can be aggregated to physical (PCS) and mental (MCS) component summary scores. Alternative scoring algorithms for correlated component scores (PCS_c_ and MCS_c_) have also been suggested. The PROMIS-29 is a newer but increasingly used HRQL measure. Analogous to the SF-36, physical and mental health summary scores can be derived from PROMIS-29 domain scores, based on a correlated factor solution. So far, scores from the PROMIS-29 are not directly comparable to SF-36 results, complicating the aggregation of research findings. Thus, our aim was to provide algorithms to convert PROMIS-29 data to well-established SF-36 component summary scores.

**Methods:**

Data from *n* = 662 participants of the Berlin Long-term Observation of Vascular Events (BeLOVE) study were used to estimate linear regression models with either PROMIS-29 domain scores or aggregated PROMIS-29 physical/mental health summary scores as predictors and SF-36 physical/mental component summary scores as outcomes. Data from a subsequent assessment point (*n* = 259) were used to evaluate the agreement between empirical and predicted SF-36 scores.

**Results:**

PROMIS-29 domain scores as well as PROMIS-29 health summary scores showed high predictive value for PCS, PCS_c_, and MCS_c_ (R^2^ ≥ 70%), and moderate predictive value for MCS (R^2^ = 57% and R^2^ = 40%, respectively). After applying the regression coefficients to new data, empirical and predicted SF-36 component summary scores were highly correlated (*r* > 0.8) for most models. Mean differences between empirical and predicted scores were negligible (|SMD|<0.1).

**Conclusions:**

This study provides easy-to-apply algorithms to convert PROMIS-29 data to well-established SF-36 physical and mental component summary scores in a cardiovascular population. Applied to new data, the agreement between empirical and predicted SF-36 scores was high. However, for SF-36 mental component summary scores, considerably better predictions were found under the correlated (MCS_c_) than under the original factor model (MCS). Additionally, as a pertinent byproduct, our study confirmed construct validity of the relatively new PROMIS-29 health summary scores in cardiology patients.

## Background

The assessment of health-related quality of life (HRQL) is becoming increasingly important when it comes to evaluating and improving healthcare in many medical fields, including cardiology [1–3]. Regulatory bodies worldwide, such as the European Medicines Agency and the U.S. Food and Drug Administration, have recommended measuring HRQL for several years to evaluate the efficacy and safety of medical treatments [4]. As a consequence, many different HRQL measurement instruments have been developed and used in patients with cardiovascular disease [5]. Since the results of different HRQL measures cannot be directly compared, the aggregation of research findings is often difficult or not possible at all [6]. Therefore, there is an urgent need for developing methods that allow to convert the scores of one HRQL measure into the scores of another [7, 8].

HRQL is a multidimensional construct most commonly assessed using patient-reported outcome measures (PROM) [9]. Over the past decades, a vast number of PROMs have been developed for the assessment of many different domains of physical and psychosocial health, such as physical functioning, pain intensity and interference, fatigue, sleep disturbance, depression, anxiety, and many more [10]. The use of such narrowly specified health domains has the advantage that outcome assessments can be adapted to specific contexts in the best possible way [11]. However, for certain research questions, it appears to be more meaningful to use composite measures that combine different aspects of HRQL into an aggregated score representing a broader health concept [12]. This may be particularly the case when a general indicator of physical or mental health is required to compare groups from different populations, or when the population of interest is heterogeneous and has a wide range of impaired HRQL domains [13].

The 36-ltem Short-Form Health Survey (SF-36), developed in the Medical Outcome Study in the early 1990s, is still one of the most frequently used generic HRQL measures [12, 14, 15]. The SF-36 consists of eight domains which can be scored separately. In addition, the individual subscale scores of each domain can be aggregated to physical (PCS) and mental (MCS) component summary scores (i.e., weighted sum scores), which are widely used composite measures of physical and mental health [15, 16]. These two distinct higher-order summary scores were derived from principal component analysis, explaining more than 80% of reliable variance of the eight SF-36 subscales [16].

Originally, PCS and MCS were derived using an orthogonal factor model, meaning that PCS and MCS were assumed to be uncorrelated when establishing scoring algorithms. This approach has some advantages over an oblique (i.e. correlated) factor model, including simplicity and straightforward interpretation of the individual scales [16]. Nonetheless, subsequent research has shown that the assumption that physical and mental health are independent constructs may not hold [17–19]. As a consequence, modified scoring algorithms for correlated SF-36 summary scores (PCS_c_ and MCS_c_) have been suggested [20]. Although many studies have shown that mental and physical health are actually quite strongly related, making correlated components more plausible than uncorrelated components [20, 21], PCS_c_ and MCS_c_ are still used less frequently than original SF-36 PCS and MCS.

The 29-item Patient-Reported Outcomes Measurement Information System (PROMIS) adult profile measure (PROMIS-29) is a newer generic measure of HRQL which is increasingly used as an alternative to the SF-36 [10, 22]. The PROMIS-29 assesses eight domains related to physical and psychosocial health, which slightly differ from the domains of the SF-36. However, the largest difference – and advantage – of the PROMIS-29 is that its items were included for original calibration of comprehensive PROMIS item banks using item response theory (IRT) methodology [23]. Thus, PROMIS-29 domain scores are placed on the same T-score metric as all other items of the corresponding domain-specific item bank. Analogous to the SF-36, physical and mental health summary scores can be derived from PROMIS-29 domain scores [22]; respective scoring algorithms are based on a correlated two-factor model representing physical and mental health [21].

Given that more and more researchers are expected to use PROMIS measures for patient-reported outcome assessments, it seems crucial to enable comparisons of respective results to other (e.g. older) studies using the SF-36. The aims of this study were therefore to establish easy-to-apply algorithms to reliably convert PROMIS-29 data to SF-36 summary component scores, and to validate these algorithms in new data not used for parameter estimation, in patients with cardiovascular disorders.

## Methods

### The Berlin long-term observation of vascular events (BeLOVE) study

The BeLOVE study is an ongoing long-term prospective observational cohort study of patients at very high risk for future cardiovascular events [24]. To meet inclusion criteria, patients must be at least 18 years of age and either recently hospitalized for an acute cardiovascular event (CVE) (acute coronary syndrome, acute heart failure, acute cerebrovascular disorder, and acute kidney injury) or at very high risk chronic cardiovascular conditions without event in the past 12 months. Pregnancy or breastfeeding, lack of health insurance, and life expectancy of ≤ 6 months due to a non-cardiovascular cause, active cancer, or a history of organ transplantation at the time of inclusion were defined as exclusion criteria. Moreover, patients unable to provide written informed consent are not considered for participation. Recruitment started in 2017 at the clinical campuses of the Charité - Universitätsmedizin Berlin and is ongoing.

The aim of BeLOVE is to improve prediction and understanding of disease progression and outcomes in patients with a very high risk of cardiovascular events, both in the acute and chronic phase, to ultimately improve and further personalize disease management. Assessments include comprehensive deep clinical and molecular phenotyping as well as ascertainment of clinical outcomes, e.g. major adverse CVEs, at predefined visits for up to 10 years.

In addition to clinical parameters, patient-reported outcome measures, including the PROMIS-29 profile and the SF-36, are administered at several assessment points of the BeLOVE study. Study data are collected and managed using REDCap [25, 26]. The present study utilized data from patients who were recruited during the first study phase of BeLOVE between July 2017 and December 2020 and had participated in the PROM collection part of the study.

### Measures

#### SF-36 physical (PCS) and mental (MCS) component summary scores

The SF-36 consists of eight domains: physical functioning (PF, 10 items), role function physical (RP, 4 items), bodily pain (BP, 2 items), general health (GH, 5 items), vitality (VT, 4 items), social functioning (SF, 2 items), role function emotional (RE, 3 items), and mental health (MH, 5 items) [14]. Scores of each domain can be transformed to a 0-100 scale.

The domain scores can be aggregated to physical (PCS) and mental (MCS) component summary scores; higher scores are representing better physical or mental health [16]. A norm-based T-score metric is used for scoring both the PCS and the MCS, with a mean of 50 and a standard deviation (SD) of 10 in the U.S. general population [16]. SF-36 PCS and MCS scores were originally derived using an orthogonal factor model, ‘forcing’ physical and mental components to be uncorrelated [16]. Since this original approach leads to potential problems with interpretation of results [17–19], modified scoring algorithms for correlated, i.e., oblique, SF-36 component summary scores (PCS_c_ and MCS_c_), have been developed [20]. In the present study, we used component summary scores from both the orthogonal and the oblique factor solution, based on the German version of the standard SF-36 instrument with original recall periods (‘the past 4 weeks’ for most items) [16, 27].

### The PROMIS-29 v2.0 profile

The PROMIS initiative, which was funded by the U.S. National Institutes of Health, developed item banks for many physical and psychosocial self-reported health domains [23]. All items of a given item bank are calibrated to a unidimensional T-score metric with a general population mean of 50 and a SD of 10, using IRT modeling [28]. A main advantage of IRT-calibrated item banks is that any item subset (e.g., short form) can be used to yield T-scores on a standardized scale [29, 30]. The PROMIS-29 v2.0 profile consists of 4-item short forms of seven HRQL domains (pain interference, fatigue, depression, anxiety, sleep disturbance, physical function, and ability to participate in social roles) and an additional single item measuring pain intensity on a 0–10 numeric rating scale [10, 21].

Analogous to the SF-36, the domains of the PROMIS-29 can be aggregated to physical and mental health summary scores, which are based on a correlated factor solution [21]; higher scores indicate better health. Many PROMIS measures have been translated into other languages, including German [31–33]. This study used the German version of the PROMIS-29 v2.0 profile [31].

### Study samples

Within the BeLOVE study, both the PROMIS-29 and the SF-36 were performed during the deep phenotyping visits ~ 90 days (visit 3, V3) and two years (visit 6, V6) after the qualifying CVE for the acute disease entities or following study inclusion in the chronic CV arm in the BeLOVE Unit of the Berlin Institute of Health at Charité - Universitätsmedizin Berlin. Because most SF-36 and PROMIS-29 domains consists of few items and to ensure stable estimates, data from participants who did not answer all items of both measures were excluded for further analysis; this approach has been applied before [20]. In the present study, we used V3 data to establish algorithms to predict SF-36 summary scores from PROMIS-29 (‘calibration sample’), while V6 data were used to validate these algorithms (‘validation sample’).

### Sample size considerations

With regard to the calibration sample, a minimum sample size of 509 was calculated to be sufficient for detecting a small effect (*f*2 > 0.02) in a linear regression model with eight predictors (power = 0.80, significance level = 0.05). A minimum sample size of 180 in the validation sample was calculated for detecting small effect sizes, defined as a standardized mean difference (SMD) of > 0.20 (power = 0.80, significance level = 0.05).

### Statistical analysis

Based on data from the calibration sample, we fitted four separate linear regression models each for predicting SF-36 physical and SF-36 mental component scores [34]. These regression models differed by both the dependent variables (uncorrelated versus correlated SF-36 component summary scores) and the predictors (PROMIS-29 domain scores versus PROMIS-29 physical/mental summary scores).

For each model, assumptions of (multiple) linear regression analysis were checked [34]. We inspected partial regression plots to rule out non-linear relationships between dependent and independent variables. To identify outliers potentially biasing the regression model, we calculated Cook’s distance values (cut-off < 1). To test the assumption of independent residuals, we used the Durbin-Watson statistic [35], which should be close to a value of 2. Homoscedasticity was checked graphically [36]. Variance Inflation Factors were calculated to rule out multicollinearity in those models with multiple predictors (cut-off < 10).

We then applied the established regression coefficients to predict SF-36 physical and mental component summary scores from PROMIS-29 data in the validation sample. Pearson correlation coefficients (r) were calculated to determine the association between empirical (i.e., ‘observed’) and predicted SF-36 summary scores. For calculating SMDs for paired samples, we utilized a pragmatic approach as described by Cumming (2012), which is appropriate for determining within-group effect sizes [37]. Specifically, we used the formula: SMD = mean difference between both measurements divided by the averaged standard deviation [37]. We considered SMD values of 0.2, 0.5, and 0.8 as small, medium, and large effects, respectively; values below 0.2 were considered negligible [38]. Mean absolute errors (mae), and root mean square errors (rmse) were used to compare the agreement between empirical and predicted scores across the different regression models [39, 40]. Smaller rmse and mae values indicate better agreement between empirical and predicted scores. Typically, the rmse is larger than the mae due to its sensitivity to larger errors.

For statistical analyses, R version 4.2.1 and the R packages ‘Metrics’, ‘effize’ and ‘pwr’ were used [40–43].

## Results

### Sample characteristics

Data from *n* = 662 and *n* = 259 patients with complete SF-36 and PROMIS 29 responses were used for calibration and validation analyses, respectively. Detailed sample characteristics with respect to age, gender, diagnosis that led to study inclusion, as well as SF-36 and PROMIS-29 scores are presented in Table 1.

**Table 1:** Sample characteristics

	Calibration sample	Validation sample
**Sample size**	662	259
**Female**; n (%)	205 (31.0)	63 (24.3)
**Mean age** (SD)	62.8 (12.2)	62.6 (11.4)
**Median age** (min.; max.)	64 (20; 88)	63 (20; 84)
**Study arm**; n (%)		
Stroke	295 (44.6)	119 (45.9)
Diabetes	168 (25.4)	51 (19.7)
Myocardial infarction	145 (21.9)	74 (28.6)
Heart failure	29 (4.4)	12 (4.6)
Acute renal failure	25 (3.8)	3 (1.1)
**SF-36 Scores**; mean (SD)		
PF	68.8 (27.0)	71.9 (27.4)
RP	56.0 (41.9)	68.2 (40.6)
BP	68.8 (27.3)	68.0 (25.2)
GH	56.8 (19.3)	59.7 (20.3)
VT	56.8 (20.1)	59.6 (20.9)
SF	76.6 (23.2)	79.9 (24.0)
RE	70.0 (40.5)	73.6 (39.7)
MH	72.9 (17.5)	75.4 (17.4)
**PCS**	**42.5 (11.2)**	**44.1 (11.5)**
**MCS**	**49.6 (10.7)**	**50.7 (11.1)**
**PCS**_**c**_	**43.5 (10.8)**	**45.4 (10.8)**
**MCS**_**c**_	**46.9 (9.7)**	**48.5 (9.8)**
**PROMIS-29 Scores**; mean (SD)		
Physical function	47.2 (8.3)	48.6 (8.3)
Fatigue	48.3 (9.3)	47.2 (9.6)
Depression	49.8 (7.9)	48.6 (7.7)
Anxiety	50.5 (7.7)	49.4 (7.7)
Sleep disturbance	49.2 (8.5)	48.5 (7.4)
Pain interference	51.7 (9.3)	51.4 (8.6)
Ability to participate	51.8 (9.0)	52.5 (9.5)
Pain intensity	51.6 (10.8)	51.6 (10.1)
**Physical summary**	**47.6 (8.6)**	**48.9 (8.6)**
**Mental summary**	**50.9 (8.1)**	**51.9 (8.1)**

*Abbreviations*: max., Maximum; min., Minimum; n, number; SD, standard deviation

SF-36 component summary scores and PROMIS-29 health summary scores indicated slightly better physical and mental health in the validation sample than in the calibration sample. However, these differences were less than 2 T-scores on a scale with a SD of about 10, corresponding to negligible effect sizes.

### Calibration of regression coefficients

Assumptions of (multiple) linear regression analysis were met for all fitted models. Table 2 summarized the regression results for both the uncorrelated (i.e., original) and correlated SF-36 component summary scores as outcomes, and with different predictors (i.e., PROMIS-29 domain score models versus PROMIS-29 summary score models).

**Table 2:** Regression results based on the calibration sample

	Dependent variables				
Regression models with different predictors (independent variables)	Uncorrelated summary scores		Correlated summary scores		
	PCS	MCS	PCS_c_	MCS_c_	
**PROMIS-29 domain scores**					
Adjusted R-squared	76.1%	56.8%	80.6%	74.0%	
Intercept	17.947*	104.129*	44.890*	82.026*	
Slopes					
Physical function ^1^	0.649*	-0.266*	0.415*	0.046	
Fatigue ^1^	-0.067	-0.198*	-0.147*	-0.210*	
Depression ^1^	0.121*	-0.476*	-0.077	-0.330*	
Anxiety ^1^	0.093*	-0.351*	-0.062	-0.233*	
Sleep disturbance^1^	-0.011	-0.118*	-0.060*	-0.104*	
Pain interference ^1^	-0.369*	0.082	-0.277*	-0.073	
Ability to participate ^1^	0.145*	0.204*	0.221*	0.202*	
Pain intensity ^2^	-0.514*	-0.030	-0.428*	-0.177	
**PROMIS-29 physical summary**					
Adjusted R-squared	70.5%	-	69.8%	-	
Intercept	-9.563*	-	-6.100*	-	
Slope	1.094*	-	1.041*	-	
**PROMIS-29 mental summary**					
Adjusted R-squared	-	40.3%	-	71.3%	
Intercept	-	6.815*	-	-4.663*	
Slope	-	0.840*	-	1.013*	

*Abbreviations*: MCS, uncorrelated Short Form-36 mental component score; MCS_c_, correlated Short Form-36 mental component score; PCS, uncorrelated Short Form-36 physical component score; PCS_c_, correlated Short Form-36 physical component score; PROMIS-29, Patient-Reported Outcomes Measurement Information

29-item profile measure v2.0

^1^ T-scores with a general population mean = 50 (standard deviation = 10) based on 4-item short forms

^2^ Single 0–10 numeric pain rating item

* Predictor is statistically significant (*p* < 0.05)

### Uncorrelated (original) SF-36 component summary scores

For predicting the SF-36 PCS, adjusted R^2^ values were high for both the PROMIS-29 domain score model (76%) and the model with the PROMIS-29 physical summary score as single predictor (71%). In the PROMIS-29 domain score model, the strongest predictors of the SF-36 PCS were physical function, pain intensity, and pain interference.

When using the PROMIS-29 to predict the SF-36 MCS, considerably less variation could be explained, compared to predicting the SF-36 PCS. In the domain score model, the adjusted R^2^ value was 57%, with depression and anxiety being the strongest predictors of the SF-36 MCS. Using the PROMIS-29 mental summary score as single predictor, only 40% of variation in the SF-36 MCS could be explained.

### Correlated SF-36 component summary scores

When using the PROMIS-29 physical health summary score for predicting the SF-36 PCS_c_, the adjusted R^2^ value was comparably high to the model with the uncorrelated PCS as outcome (70%). In the multiple regression model with individual PROMIS-29 domain scores as predictors, even more variation of the PCS_c_ could be explained (adjusted R^2^ = 81%), with physical function and pain intensity being the strongest predictors.

For predicting the SF-36 MCS_c_, the adjusted R^2^ value was also high for both the PROMIS-29 domain score model (74%) and the model with the PROMIS-29 mental health summary score as single predictor (71%). In the PROMIS-29 domain score model, the strongest single predictor of the SF-36 MCS_c_ was depression.

### Validation of regression models

#### Uncorrelated (original) SF-36 component summary scores

**Fig. 1:**
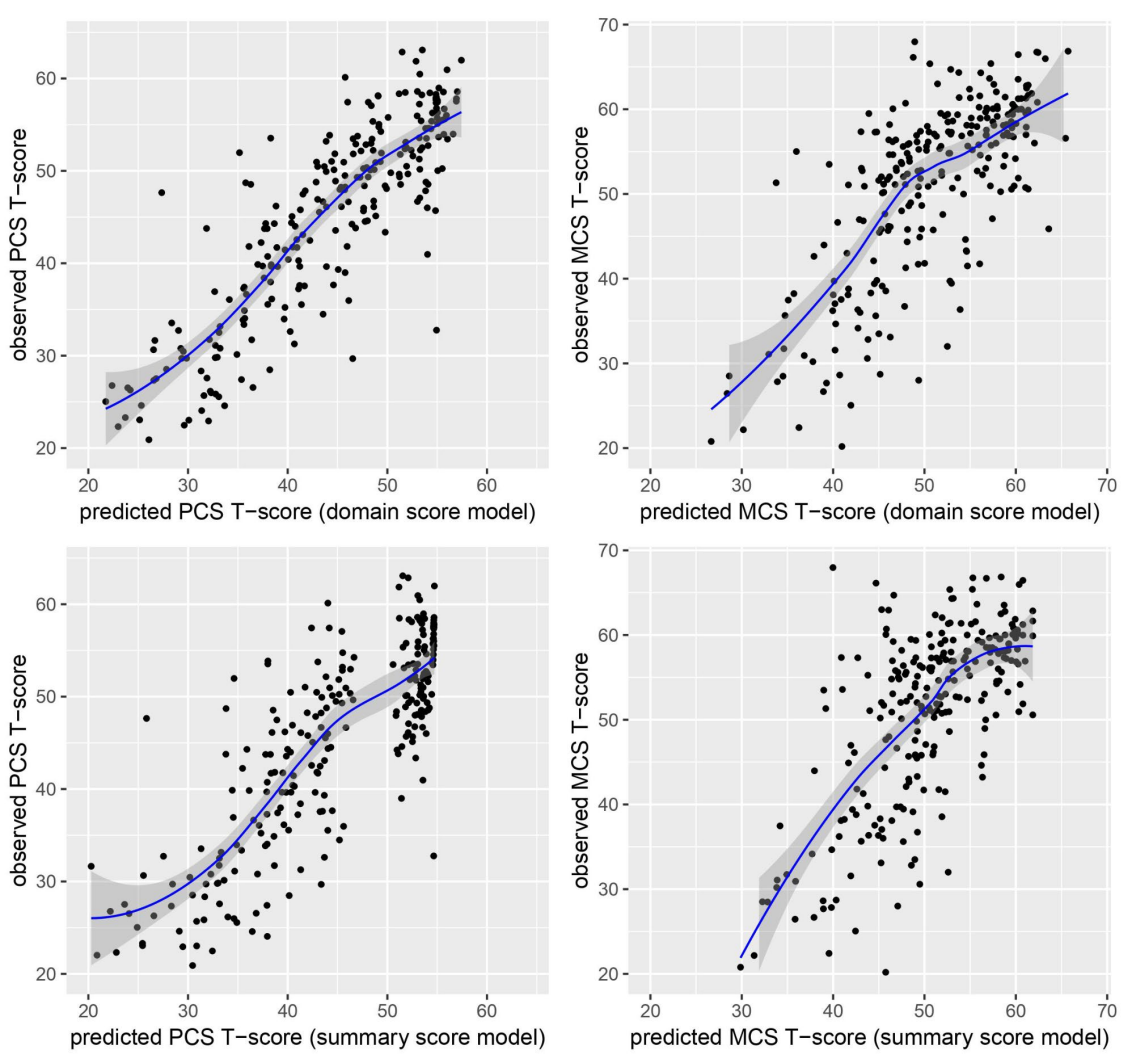
Scatter plots showing the associations between predicted (x-axis) and observed (y-axis) SF-36 component summary scores (uncorrelated model)

Results of applying the previously established regression coefficients to predict original SF-36 PCS and MCS scores from PROMIS-29 data in the validation sample are presented in Table 3. Pearson correlation coefficients between empirical and predicted SF-36 PCS scores were high, with *r* = 0.87 for the PROMIS-29 domain score model and *r* = 0.83 for the PROMIS-29 summary score model. With regard to predicting SF-36 MCS scores, the association between empirical and predicted scores were lower, with *r* = 0.75 for the PROMIS-29 domain score model and *r* = 0.68 for the PROMIS-29 summary score model. Related scatter plots are presented in Fig. 1, showing that predicted scores appear to be generally less biased in the domain score model as compared to the summary score models. Predicted PCS scores based on PROMIS-29 summary scores indicated ceiling effects.

**Table 3:** Validation results for uncorrelated (original) SF-36 component summary scores

Statistics	PCS		MCS	
	Empirical	Predicted	Empirical	Predicted
PROMIS-29 domain score model				
Mean (SD)	44.1 (11.5)	43.5 (9.5)	50.7 (11.1)	50.5 (8.1)
Pearson correlation	0.87		0.75	
SMD [95% CI]	-0.06 [-0.12, 0.01]		-0.02 [-0.11, 0.07]	
rmse	5.68		7.44	
mae	4.33		5.76	
**PROMIS-29 summary score model**				
Mean (SD)	44.1 (11.5)	44.0 (9.4)	50.7 (11.1)	50.4 (6.8)
Pearson correlation	0.83		0.68	
SMD [95% CI]	-0.02 [-0.09, 0.05]		-0.03 [-0.13, 0.07]	
rmse	6.44		8.20	
mae	4.97		6.23	

*Abbreviations*: CI, confidence interval; MCS, uncorrelated Short Form-36 mental component score; mae, mean absolute error; PCS, uncorrelated Short Form-36 physical component score; PROMIS-29, Patient-Reported Outcomes Measurement Information 29-item profile measure v2.0; rmse, root mean square error; SD, standard deviation; SMD, standardized mean difference

On the group level, empirical and predicted SF-36 summary scores differed only negligible, which is true for the PCS and the MCS, and for both the PROMIS-29 domain score model and the PROMIS-29 summary score model (SMDs between -0.06 and -0.02). However, the agreement between empirical and predicted summary scores, as assessed with the rmse and the mae, was better for predicting the PCS than the MCS.

### Correlated SF-36 component summary scores

**Fig. 2:**
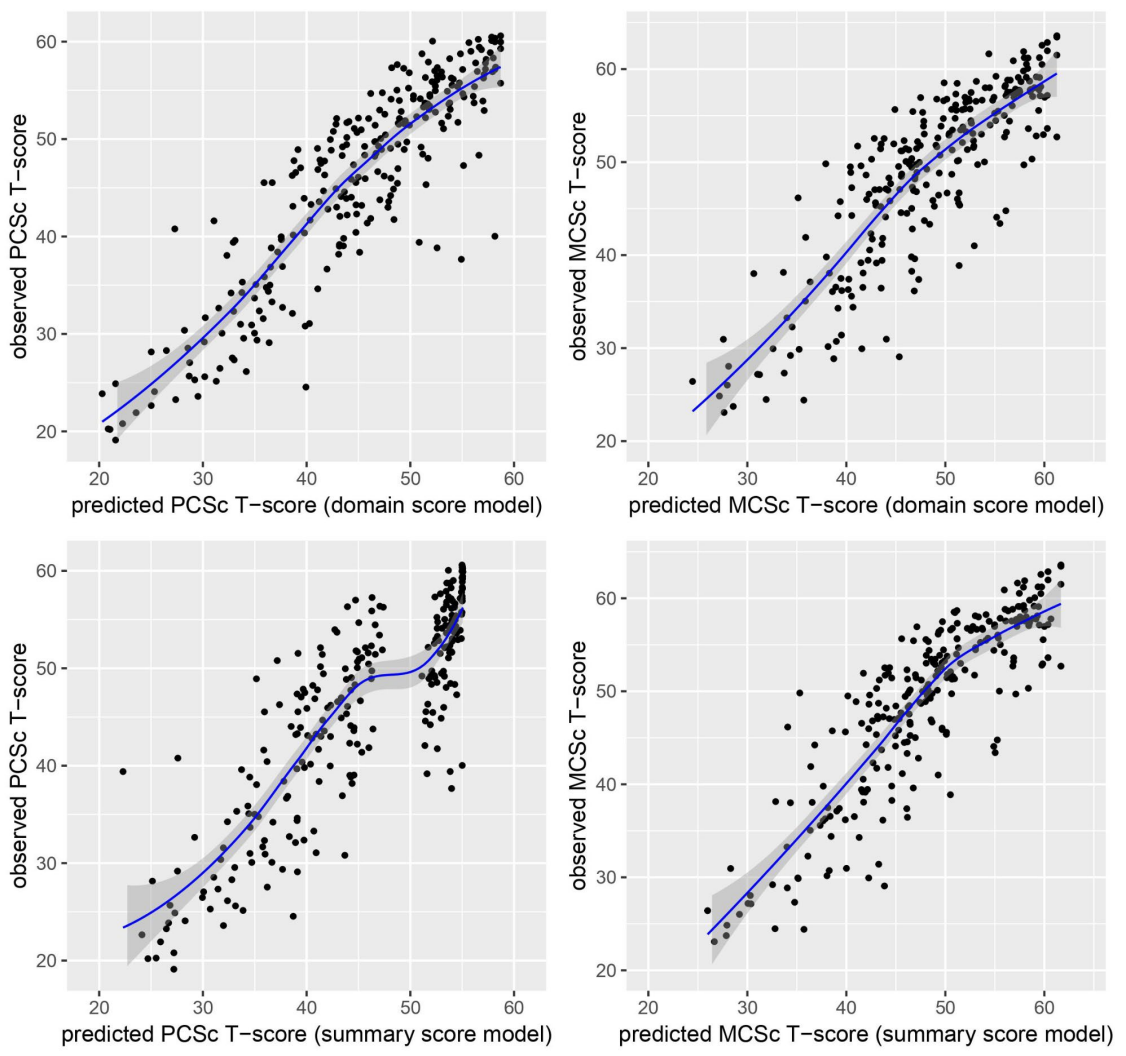
Scatter plots of the validation data showing the associations between predicted (x-axis) and observed (y-axis) SF-36 component summary scores (correlated model)

Measurement characteristics after applying the established regression coefficients to predict correlated SF-36 PCS_c_ and MCS_c_ scores from PROMIS-29 data in the validation sample are presented in Table 4. Pearson correlation coefficients between empirical and predicted SF-36 component summary scores were generally high, with *r* ≥ 0.85 for all regression models. Scatter plots again indicated ceiling effects, when PROMIS-29 summary scores were used to predict PCS_c_ scores (see Fig. 2).

**Table 4:** Validation results for correlated SF-36 component summary scores

Statistics	PCS_c_		MCS_c_	
	Empirical	Predicted	Empirical	Predicted
PROMIS-29 domain score model				
Mean (SD)	45.4 (10.8)	44.6 (9.4)	48.5 (9.8)	48.1 (8.3)
Pearson correlation	0.90		0.86	
SMD [95% CI]	-0.07 [-0.13, -0.02]		-0.03 [-0.12, 0.01]	
rmse	4.82		5.03	
mae	3.74		3.99	
**PROMIS-29 summary score model**				
Mean (SD)	45.4 (10.8)	44.8 (8.9)	48.5 (9.8)	47.9 (8.2)
Pearson correlation	0.85		0.87	
SMD [95% CI]	-0.05 [-0.12, 0.01]		-0.06 [-0.10, 0.03]	
rmse	5.68		4.90	
mae	4.53		3.90	

*Abbreviations*: CI, confidence interval; MCS_c_, correlated Short Form-36 mental component score; mae, mean absolute error; PCS_c_, correlated Short Form-36 physical component score; PROMIS-29, Patient-Reported Outcomes Measurement Information 29-item profile measure v2.0; rmse, root mean square error; SD, standard deviation; SMD standardized mean difference

The differences between empirical and predicted SF-36 summary score on the group level were negligible in each model (SMDs between -0.07 and -0.03). In contrast to the regression models with uncorrelated SF-36 PCS and MCS scores, the agreement between empirical and predicted summary scores, as assessed with the rmse and the mae, was comparably high between models with PCS_c_ and MCS_c_ scores as outcomes.

## Discussion

Based on a sample from a large cardiovascular cohort, we established and validated regression coefficients that can be used to convert PROMIS-29 data to SF-36 physical and mental component summary scores. Satisfactory model fit was confirmed by applying the regression coefficients to new data from a subsequent observation of the same cohort, supporting validity of the established linear regression models in patients with cardiovascular diseases.

We found that using all eight PROMIS-29 domains to predict SF-36 component summary scores tended to produce slightly better results than using PROMIS-29 health summary scores as single predictors. Thus, if PROMIS-29 domain scores are available, we recommend using them directly to estimate SF-36 scores and avoid the intermediate step of calculating PROMIS-29 health summary scores. However, if only summary scores are available, they can also be used as reliable predictors.

Regarding the prediction of SF-36 physical component summary scores, we found comparable results for either the original (PCS) or the correlated factor model (PCS_c_). The predictive power of PROMIS-29 scores was high for both the PCS and the PCS_c_. In the PROMIS-29 domain score models, the pattern of individual predictors was very similar, with physical function and pain being the strongest predictors for PCS and PCS_c_ scores.

In contrast, for predicting SF-36 mental component summary scores using the PROMIS-29, considerably better predictive power and less biased predictions were found under the correlated (MCS_c_) than under the original factor model (MCS). A potential reason for this is that SF-36 component summary scores under an orthogonal (i.e., uncorrelated) factor model might be biased, which has been discussed before [17–20]. For example, we found that low PROMIS physical function scores were significantly associated with higher MCS scores, which does not seem plausible. For predicting MCS_c_ scores based on individual PROMIS-29 domains, this was not the case. A further reason for the particularly high association between PROMIS-29 mental summary scores and SF-36 MSC_c_ scores might be that PROMIS health summary scores were also established under a correlated factor model [21].

This study has limitations. First, the representativeness of our sample might be affected by self-selection since patients in the cohort refused to participate in the PROM part of the study. Unfortunately, we do not know how the sample used for analysis differs from the full cohort with regard to self-reported health. However, both PROMIS-29 and SF-36 scores covered the full range of possible T-scores, indicating that the established regression coefficients are reliable for subpopulations with differing health status. A second limitation is that our study is based on data from German patients with cardiovascular diseases. It remains to be investigated whether our results can be generalized to non-German populations and to populations with non-cardiovascular disorders. Third, PROMIS-29 physical health summary scores showed ceiling effects, supporting the recommendation to preferably use individual PROMIS-29 domain scores for predicting SF-36 summary component scores. A fourth limitation is that many alternative methods for mapping scales are available [8, 44–46], which we did not employ. We chose linear regression for its simplicity, making our algorithms accessible to a broad audience within a well-known framework. Moreover, we consistently included all PROMIS-29 domains in our multiple regression models (and avoided stepwise selection of predictors [7]) to maintain consistency with the algorithms used for calculating PROMIS and SF-36 component scores [16, 20, 21]. In this context, we also experimented with polynomial regression coefficients [7], which did not yield notable improvements in predictive power (data not shown) compared to the linear models. Furthermore, we did not employ bidirectional mapping methods, such as equipercentile equating or IRT-based linking methods [8, 44, 45], as our aim was to used PROMIS data on domain-level to predict SF-36 composite scores (which was empirically supported by yielding superior results compared to using PROMIS composite scores as predictors).

Despite these limitations, the results of this study are of practical importance for future research, particularly for measuring overall self-reported HRQL in cardiovascular populations. Measures of overall HRQL might be advantageous over the use of individual health domains in some research contexts [21]. Moreover, the number of statistical comparisons, e.g., in studies of treatment efficacy, can be reduced by using health component scores [13, 16]. The SF-36 is probably the most frequently used generic measure of self-reported HRQL. However, the PROMIS-29, which is a newer measure based on modern test theory methods, has been discussed to be even more appropriate for assessing HRQL component scores than the SF-36 [47]. A particular advantage of the flexible PROMIS approach is that any item set of a given domain can be used to establish domain-related T-scores [23]. Consequently, PROMIS-29 health summary scores can also be produced based on other items than those included in the PROMIS-29 profile measure, as long as T-score estimates for each of its eight domains can be provided.

In this context, by showing high associations with SF-36 component summary scores, the findings of our study confirmed construct validity of PROMIS-29 health summary scores. In view of these advantages, we expect PROMIS to be increasingly used for HRQL assessments, highlighting the usefulness of valid comparisons to research findings based on the SF-36.

## Conclusions

In sum, this study will help facilitating comparison and pooling of findings from the SF-36 and the PROMIS-29 profile, two of the most frequently used generic measures of self-reported HRQL. We hope that our study will encourage other researchers to replicate our models in other patient populations.

## Data Availability

The BeLOVE consortium aims to ensure that the collected data and sample material will be used for the greatest possible benefit to health-related research, in particular cardiovascular research. Researchers interested in the data of BeLOVE may apply for data access through our use and access committee, as long as one member of the project team is part of the BIH research community to support the research process. The use and access committee evaluates the merits and technical feasibility of the project proposal and assesses potential overlap with ongoing projects and analyses. Data transfer will be performed according to established General Data Protection Regulation (GDPR) data sharing guidelines.

## Abbreviations

BeLOVE: Berlin Long-term Observation of Vascular Events
BP: Bodily Pain
CVE: Cardiovascular Event
GH: General Health
HRQL: Health-Related Quality of Life
IRT: Item Response Theory
mae: Mean Absolute Error
MCS_c_: Correlated Mental Component Summary Scores
MCS: Original Mental Component Summary Scores
MH: Mental Health
PCS_c_: Correlated Physical Component Summary Scores
PCS: Original Physical Component Summary Scores
PF: Physical Functioning
PROM: Patient-Reported Outcome Measure
PROMIS: Patient-Reported Outcomes Measurement Information System
RE: Role Function Emotional
rmse: Root Mean Square Error
RP: Role Function Physical
SD: Standard Deviation
SF: Social Functioning
SMD: Standardized Mean Difference
VT: Vitality
